# Literary Notes

**Published:** 1900-08

**Authors:** 


### LITERARY NOTES.

An Appendix to the International Directory of Laryngologists and Otologists, compiled by Mr. Richard Lake, is in course of preparation. In it will be found corrections of names and addresses already given, an additional list of names and addresses received since publication, and an obituary list.

. . . . Considerable additions have been obtained for the foreign list, which will materially add to its value and completeness.

The decision of the Editors of the *Journal of Laryngology, Rhinology, and Otology*, under whose auspices the Directory is published, to allow no name to be inserted in the British list for which sanction has not been given in writing, at once explains some omissions and criticisms. The editors, whilst desirous of making the directory as complete as possible, consider it best to adhere to this course. It is therefore hoped that all engaged in the practice of laryngology, rhinology and otology will assist as far as possible in making this useful work complete, by sending in their names and addresses to the Editor, International Directory of Laryngologists and Otologists, 129, Shaftesbury Avenue, London, W.C., Eng.—*Journal of Laryngology, Rhinology, and Otology*, April, 1900.

---

HOWARD CHALLEN, of 150 Nassau Street, New York, is arranging an exhaustive list of periodicals, medical books, etc., for ready reference. Mr. Challen is the publisher, also, of registers for the use of publishers, such as subscription and advertisers' records, as well as other labor-saving books of a similar kind.

---

WARNER'S new therapeutic reference book is one of the very few guides of its kind now offered to students and busy practitioners, and must not be confused with Warner's Therapeutic Reference Book. The latter has been discarded, the new one taking its place. As its preface states it is not intended to teach graduates anything about therapeutics, but it is to be regarded rather as a handy aid to the memory. Many valuable tables are represented, including the metric table, thermometric equivalents, etc., useful tests for various matters, including urinary tests for albumin, sugar, etc., comparative values of certain goods, a complete dose table of drugs, a list of diseases and their remedies, hints as to indications of pregnancy, recommendations as to post mortem examinations, etc., etc. The brief mention above gives a suggestion of the many valuable departments of this new book.

It is bound in two styles, one leather, at 50 cents, and the other leatherette, at 25 cents per copy, postage prepaid in both instances. Address the publishers, William R. Warner & Co., Philadelphia, Pa.

---

THE Essentials of Hematology is the title of a handsomely printed brochure recently published by the Palisade Manufacturing Company, of Yonkers, N.Y. Its purpose is to present a brief review of the actual status of blood work as well as a concise and practical guide for a physician who desires to do a little work on his own account. It gives a description of the needed instruments, their methods of use and a few simple ways of staining. Then follows a brief description of the blood in primary and secondary anemia, leukemia, and malaria, to which is added a chapter on Widal's test for typhoid fever. It is handsomely illustrated, contains much useful information in condensed form and will be supplied on application to the publishers.

---

MOSQUITOES and Malaria is the title of a pamphlet published by McKesson & Robbins, of New York. Its object is to set forth the rôle played by the mosquito in the transmission of malarial fever. It gives much useful information in concise form, is well illustrated and will be furnished by the publishers upon application.

---

THE twenty-fourth year book of the United States Reformatory at Elmira for the fiscal year ending September 30, 1899, is a well printed brochure of 109 pages. It contains the reports of the several officers of the institution, a few illustrations, and in editing, typography, illustration and binding is solely the product of prisoners' labor.

